# The coevolution between APOBEC3 and retrotransposons in primates

**DOI:** 10.1186/s13100-022-00283-1

**Published:** 2022-11-29

**Authors:** Giorgia Modenini, Paolo Abondio, Alessio Boattini

**Affiliations:** 1 Department of Biological, Geological and Environmental Sciences, University of Bologna, Bologna, Italy; 2 Department of Cultural Heritage, University of Bologna, Ravenna, Italy

**Keywords:** APOBEC3, Retrotransposons, Primates, Homo, Brain diseases, Evolutionary arms race, Embryonic development

## Abstract

Retrotransposons are genetic elements with the ability to replicate in the genome using reverse transcriptase: they have been associated with the development of different biological structures, such as the Central Nervous System (CNS), and their high mutagenic potential has been linked to various diseases, including cancer and neurological disorders. Throughout evolution and over time, Primates and *Homo* had to cope with infections from viruses and bacteria, and also with endogenous retroelements. Therefore, host genomes have evolved numerous methods to counteract the activity of endogenous and exogenous pathogens, and the APOBEC3 family of mutators is a prime example of a defensive mechanism in this context.

In most Primates, there are seven members of the APOBEC3 family of deaminase proteins: among their functions, there is the ability to inhibit the mobilization of retrotransposons and the functionality of viruses. The evolution of the APOBEC3 proteins found in Primates is correlated with the expansion of two major families of retrotransposons, i.e. ERV and LINE-1.

In this review, we will discuss how the rapid expansion of the APOBEC3 family is linked to the evolution of retrotransposons, highlighting the strong evolutionary arms race that characterized the history of APOBEC3s and endogenous retroelements in Primates. Moreover, the possible role of this relationship will be assessed in the context of embryonic development and brain-associated diseases.

## Introduction

Located on human chromosome 22, the APOBEC3 (apolipoprotein B mRNA-editing catalytic polypeptide-like 3) genes encode for deaminase proteins that can catalyze the deamination of cytosine-to-uracil (C to U) on single-stranded DNA and/or RNA. APOBEC3s (A3s) are only present in placental mammals [1, 2] and are part of the AID/APOBEC superfamily of proteins involved in immunity, metabolism, and infectious diseases (reviewed in [3]). In most primates and *Homo*, the APOBEC3 family is represented by seven members: APOBEC3A/B/C/D/F/G/H, first annotated by Jarmuz et al. (2002) [4].

Since their discovery, A3 genes have been studied mostly for their capacity of inhibiting a wide range of exogenous viruses, such as Human/Simian immunodeficiency virus (HIV/SIV) [5, 6] and hepatitis B virus (HBV) [7]. In humans, four genes (A3D/F/G and stable haplotypes of A3H) can inhibit HIV-1 replication by inducing C-to-U hypermutations in viral genomes and/or by deaminase-independent mechanisms [8–10].

On the other hand, A3s can counteract the mobilization of endogenous retroviruses and other retrotransposons, such as Alu and LINE-1. Indeed, retrotransposons account for nearly half of the primates’ genome, with LINEs and SINEs far more represented than ERVs, and have been constantly influencing primate genome’s evolution.

Interestingly, in primates and *Homo* A3 proteins have been faced with strong positive selection, duplications and fusions that gave rise to the currently known seven members of the APOBEC3 gene cluster. Such expansion is a consequence of the co-evolution between A3 proteins and their counterparts, i.e. viruses and retrotransposons [11, 12].

In this review, we will discuss the state-of-the-art literature about the evolution of A3 genes and retrotransposons, focusing on the role of the formers in regulating mobilization and expression of endogenous retroelements. Finally, we will highlight the strong evolutionary link between A3 proteins and retrotransposons, which probably co-evolved in the context of a strong evolutionary arms race that characterized the patterns of speciation, radiation and evolution of primates.

### Overview of retrotransposons

Transposable Elements (TEs), discovered in the mid-1940s by Barbara McClintock [13], are short DNA sequences, usually between a few hundred bp and ~ 10 kb [14–19] (but polintons can be longer than 20 kb [20–22]), which have the ability to replicate or multiply in the genome.

**Fig. 1:**
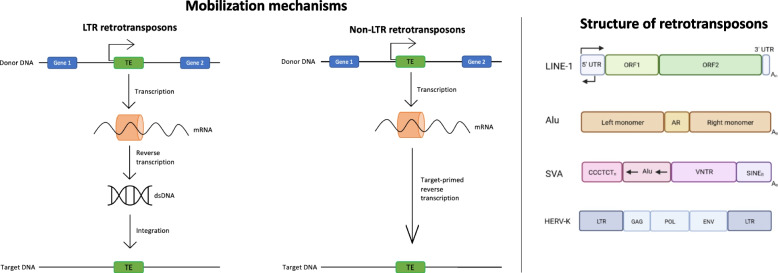
Retrotransposons mobilization mechanisms and structure. Non-LTR retrotransposons move via an RNA intermediate that is then reverse-transcribed

Retrotransposons, which belong to Class I mobile elements, move via an RNA intermediate [23, 24] that is then reverse-transcribed and use a *copy-and-paste* mechanism that allows these elements to increase the number of their copies. They are grouped into Long Terminal Repeats retrotransposons, to which the Human Endogenous Retrovirus (HERV) family belongs, and non-LTR retrotransposons, which include Long Interspersed Elements 1 (LINE-1 s or L1s), Short Interspersed Elements (SINEs)—such as Alu-like TEs—and SINE-VNTR-Alus (SVAs) [25], as shown in Fig. 1.

HERVs are endogenous viral elements that resemble and are derived from infectious retroviruses, however they are typically not infectious. HERVs are composed of group-associated antigen (*gag*), polymerase (*pol*) and envelope (*env*) genes, along with two LTRs at the 3’ and 5’ regions [14, 15].

Full-length human LINE-1s are ~ 6 kb long [16, 17] with a ~ 900 bp long 5’ untranslated region (5’ UTR) with internal promoter activity [26], a ~ 150 bp long 3’ UTR and a poly(A) tail [16]. L1s also contain two Open Reading Frames (ORF1 and ORF2), which encode, respectively, for a ~ 40 kDa protein with RNA binding and chaperone activities [27, 28] and for a ~ 150 kDa protein with reverse transcriptase (RT) and endonuclease (EN) activities [29, 30]. Both ORFs are required for L1s mobilization in the human genome [31].

The main family of SINEs in the human genome is essentially represented by Alus. Alu elements are ~ 300 bp long and have a dimeric structure determined by the fusion of two 7SL-RNA-derived monomers, separated from each other by an A-rich linker region [32]. The 5’ region carries an internal RNA III polymerase promoter, and at the end of the element there is an oligo dA-rich tail of variable length.

SVAs are primate-specific retrotransposons that terminate with a poly-A tail (similarly to L1s). Their name synthesizes the three components of their sequence: the 3’ LTR region of the endogenous retrovirus HERV-K10 (SINE-R), a Variable Number Tandem Repeats (VNTR) region and an antisense Alu-like region. Because of the polymorphism of their VNTR region copy number (48–2.306 bp), SVAs may vary in size; however, more than a half are ~ 2 kb long [19].

L1s are the only known autonomously active TEs in humans [33–35]; on the other hand, retrotransposition in Alus and SVAs is still made possible thanks to the L1s’ enzymatic machinery [36].

### Evolution of retrotransposons in primates

TEs had a role in growing the size of Eukaryotes’ genomes [36]. In mammals, the repetitive portion is dominated by LINEs and SINEs, followed by LTR retrotransposons, and then DNA transposons. In particular, in most mammals, ~ 75% of repetitive sequences are derived from non-LTR retrotransposons [37].

In primates, approximately 50% of the genome consists of TEs. LINEs and SINEs together make up for ~ 60% of total TE sequences in all investigated species of primates, suggesting their evolutionary importance across simians and prosimians [38].

**Fig. 2:**
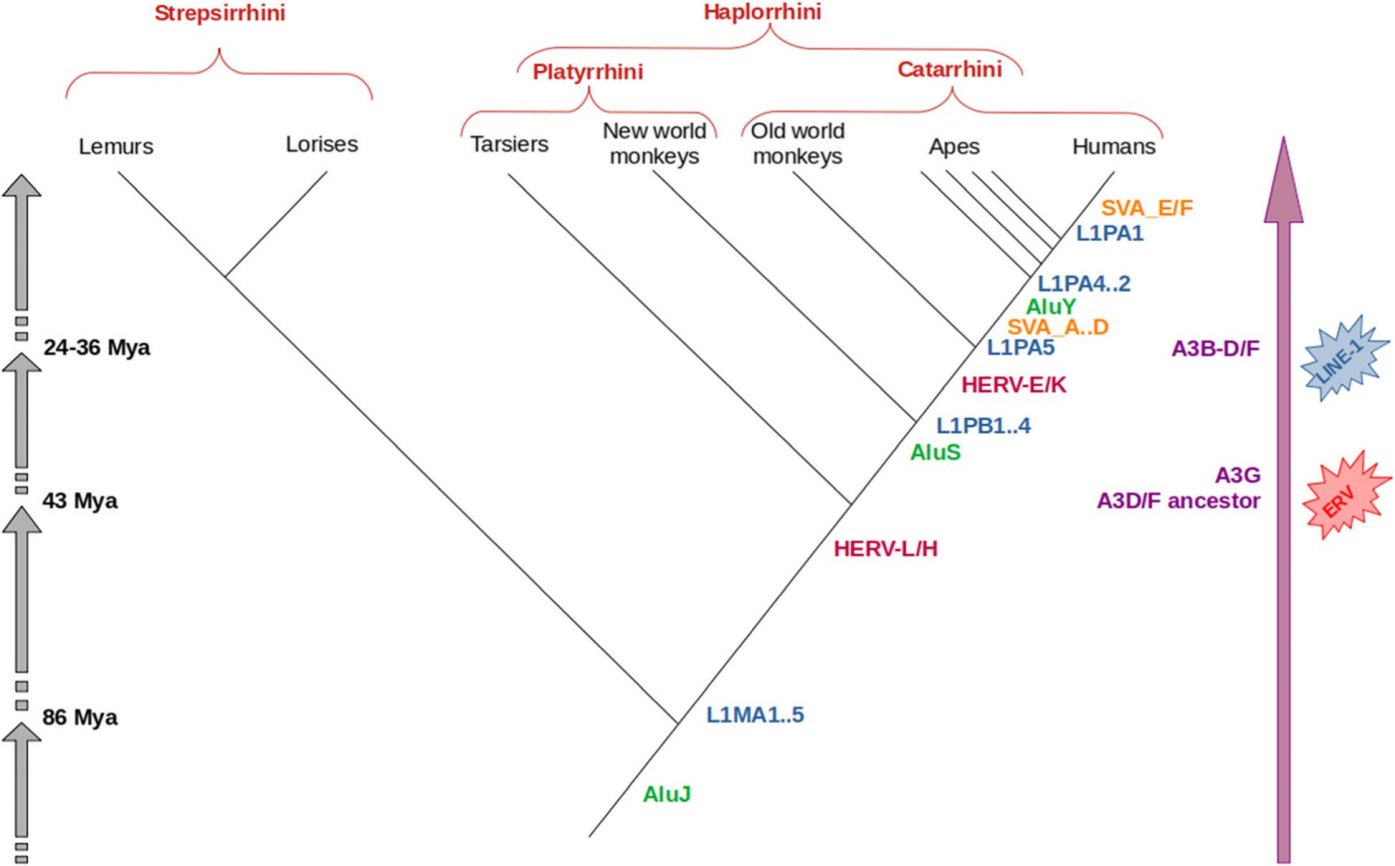
Evolutionary tree of Primates and retrotransposons. SVA elements are Hominoidea-specific, while Alu and L1 are more ancient. The origin of different APOBEC3 genes is concurrent with the explosion of specific retrotransposon families, i.e. ERVs and L1s: A3G appeared just after the split of Simiiformes 43 Mya and during the invasion of ERVs, while A3B and A3D/F originated during the invasion of LINE-1 and the split between old world monkeys and Hominoidea (Apes + Humans)

For instance, primate specific Alu elements appear to have been inserted after the radiation between prosimians and simians, approximately 100 million years ago (Mya), and a major expansion was estimated to have occurred from 50 to 25 Mya [39]. AluJ predates the division between Strepsirrhini and Haplorrhini (~ 86 Mya) and, before the divergence of Platyrrhini and Catarrhini, AluS derived from AluJ and successively took over amplification approximately 55 Mya. Finally, AluY, the youngest family, evolved from the AluS subfamily and expanded in the Catarrhine lineage (Fig. 2), with Ya5 and Yb8 dominating in humans.

The history of LINE-1 s is far less characterized: certainly, in early primate evolution as many as three L1 lineages (L1MA, L1PB, and L1PA) have been active in parallel for up to 30 My [40]. L1PA succeeded and remained active within the anthropoid lineage leading to the human specific L1PA1 [41]. Nowadays, the most active L1 subfamily in the human genome is L1-Ta1 [42], however some pre-Ta elements are still capable of retrotransposition [43, 44].

SVA elements are more recent than L1 and Alu: the lack of SVAs in old world monkeys suggests that SVAs are hominid specific retroelements [19]. SVAs are represented by seven subfamilies, named SVA_A-F. Subfamily age estimates based upon nucleotide divergence indicate that the expansion of four SVA subfamilies (SVA_A, SVA_B, SVA_C and SVA_D) began before the divergence of human, chimpanzee, and gorilla, while subfamilies SVA_E and SVA_F are restricted to the human lineage [19]. SVAs expanded in great apes, with a total of 2.700 elements in humans and around 1.800–2.500 SVA elements found in the orangutan [45] and chimpanzee [46] genomes, respectively. Alongside with SVAs, other composite elements have been identified in gibbons: LAVA (L1-Alu-VNTR-Alu), PVA (PTGR2-VNTR-Alu) and FVA (FRAM-VNTR-Alu) [47, 48]. They combine simple repeats, Alu fragments, a VNTR and variable 3' domains, which are, except for PVA, derived from other retrotransposons [49]. Proliferation of non-autonomous retrotransposons in a particular genome is dependent on their expression in the germline and/or early embryo and on their efficient interaction with the proteins synthesized from their autonomous partner [49]. Notably, the central domain of VNTR composites evolved in a lineage-specific manner which gave rise to distinct structures in gibbon LAVA, orangutan SVA, and human/chimpanzee SVA [50], suggesting an inextricable link between TEs and primate genomes that lead to speciation, radiation and evolution of primates [23].

The most ancient HERV groups formed before the separation of Catarrhini and Platyrrhini, that occurred ~ 40 Mya, being thus shared between primate species of both parvorders, as in the case of HERV-L and HERV-H. Many other HERV groups, such as HERV-E and HERV-K(HML-2), are evolutionarily younger and have been acquired after the separation of Catarrhini and Platyrrhini [51]. Upon entering the host gene pool through integration in germline cells or in the precursors of germline cells, a provirus is known as an endogenous retrovirus (ERV) and is fated for either loss or fixation depending on random genetic drift and natural selection [52]. ERVs are genetic loci whose ultimate origins trace back to exogenously replicating retroviruses, even if the vast majority of ERVs are defective for viral gene expression as a consequence of mutations accumulated across thousands to millions of years of vertebrate evolution [52].

In human genomes, current estimates of TE content range from 49 to 69% [53]. HERVs account for 5–8% of the human genome [54], and LINE-1 s are probably the most impactful TEs in humans: LINE-1-derived sequences account for ~ 17% of human genome [55] and their encoded proteins (ORF1p and ORF2p) are able to mobilize non autonomous retrotransposons, other non-coding RNAs and mRNAs, leading to the creation of processed pseudogenes. L1s and Alus together account for 60% of all interspersed repeat sequences in humans. L1s, in particular, have been identified as the TE type most active in mammals, suggesting an inextricable link between L1s and their hosts [56].

Evolutionary mechanisms such as natural selection and stochastic processes influence both the rate of fixation and frequency distribution of TEs in every organism. The efficiency of selection depends on the effective population size, which has been estimated at ~ 10^4^ in humans [57]; therefore, in our species TE insertions (both positive and deleterious) may accumulate. Indeed, most of the actual human TE insertions are remnants of ancient insertions [36].

Active retrotransposition can provide opportunities for exaptation events, build novel regulatory networks, and even increase the adaptive potential of a population (reviewed in [58–60]). Despite these benefits, many insertions are neutral or deleterious. Highly deleterious insertions will be rapidly purged from the gene pool and, thus, mammalian genomes have evolved several defense mechanisms to limit TEs expression and mitigate the potential deleterious effects of TEs activity [37], such as APOBEC3 proteins.

### Evolution of APOBEC3 family in primates

APOBEC3A/C/H have a single cytosine deaminase (CD) domain. By contrast, APOBEC3B/D/F/G have two CD domains, of which only the C-terminal CD2 is catalytically active [61]. All A3 proteins share at least one zinc (Z)-coordinating catalytic motif, and A3 genes possess either one or two conserved zinc-coordinating motifs, in which the zinc is coordinated by a histidine and two cysteines. Z motifs can be classified into three groups (Z1, Z2, Z3), all sharing the consensus amino acid signature His-X-Glu-X_23–28_-Pro-Cys-X_2–4_-Cys (where X can be nearly any residue) [3, 4, 62, 63].

The existence of three paralog zinc-coordinating motifs in the sequence of the seven APOBEC3 members in the primate lineage suggests a complex sequence of duplications and fusions that gave origin to the current ensemble of mutator proteins [12, 63, 64]. Specifically, primates carry three Z1 paralogs, seven Z2 paralogs, and one Z3 paralog distributed across the APOBEC3 gene locus on chromosome 22 [65]. In modern humans, these eleven A3 open reading frames contribute to the seven genes by encoding either a single Z domain or a fusion of two (A3Z2-A3Z2 or A3Z2-A3Z1) in a complex organization [63]. These three motifs certainly existed at least as far back as the separation between placental mammals and marsupials, 148 Mya, and may have originated from a single gene copy, possibly predating egg-laying mammals (247 Mya) [63]. Moreover, Münk and colleagues show that most duplications and rearrangements in the Z1 and Z2 groups, especially for the primate lineage, have happened over the last 100 My. When compared with their sister group, the AICDA genes, the Z groups all show a higher evolutionary rate (AICDA: 7.41 × 10^–4^ substitutions per site per My; A3s: 2 × 10^–3^ substitutions per site per My), but there is a significant decrease in the evolutionary rate of the Z groups over the last 100 My (*p*-value < 0.0007). Therefore, the A3 genes have a higher rate of substitutions than their sister groups, but the same rate has steadily reduced over time. The Z1 group has split twice: once around the basal divergence of primates (around 75 Mya), and again around the origin of the Hominoidea lineage (26 to 34 Mya) [63]. The phylogenetic relationships of the Z2 group are more complex to reveal, especially with regards to the primate lineage, but Münk and colleagues argue that a first duplication event (or even two) may have happened around the separation between Haplorrhini and Strepsirrhini (86 Mya) and certainly before the diversification of the Simiiformes (43 Mya); based on sequence similarity, the several copies of Z2 that can be found in humans have definitely appeared by duplication, but their phylogeny is intricate and separation estimates could not be clearly supported [63]. Recently, Uriu and colleagues have performed a complete reannotation of the APOBEC gene family in primates, specifically highlighting the phylogenetic subclassification of the A3 zinc domains [64]. Their work confirmed the amplification of the Z1 and Z2 domains in this lineage, together with an accelerated increase in diversification and complexity over time, especially with respect to Z3. By comparing sequences of Prosimians, New World Monkeys (NWM), Old World Monkeys (OWM) and Hominoidea, they suggest that the Z3 domain was preserved in the Simiiformes but lost in the Prosimians, while the generation of genes with multiple catalytic domains that have been conserved up to the present has first occurred in the common ancestor of Simiiformes [64]. Repeated instances of amplification, duplication and gene conversion have, then, produced the variety of A3 genes that can be observed across Simiiformes today. Interestingly, these events have been accompanied by the peak invasions of mobile elements in human DNA: specifically, ERVs peaked around the origin of A3G in the common ancestor of the Simiiformes, while LINE1 peaked around the origin of A3B, D and F in the Catarrhini clade (OWMs and Hominoidea) [64]. Ito and colleagues (2020) explored the relationship between intact A3Z domains and ERV insertions in the mammalian genome and highlighted an acceleration in the accumulation of Z domains over an increase of ERV insertions in primates [12]. At the same time, they suggested a parallel increase in the quantity of G-to-A mutations in primate ERV sequences and a higher estimated proportion of ERV insertions in the ancestor of Simiiformes, which was not subsequently carried on in the NWMs [12]. Moreover, sequence analysis allowed to detect residue conservation in the catalytic domains across all Z groups, as well as specific amino acid residues that are characteristic of each group [64]. These observations suggest a notable relationship between primate evolutionary radiation, proportion of transposable element insertions over time and amplification of the defensive repertoire that brought to the variety of A3 genes observable in our species.

### Overview of APOBEC3 functions

A3 genes are involved in various functions, from viral and retrotransposon restriction to cancer progression [66]. Indeed, several recent studies have described the role and mechanisms of action for this protein family in the context of cancer-related DNA mutagenesis, as it is becoming more and more clear that prevalent signatures of instability in cancer cell genomes are due to APOBEC3 activity on transiently exposed single-strand DNA (for example, during DNA mismatch repair and lagging strand replication) [67–72]. This activity leaves signatures along the double helix that are clearly traceable to A3 family members and are found predominantly in cancer cells [73]. As the structural details of A3s interaction with nucleic acids are being unveiled [74], the ambivalent effect of these protective enzymes is also being highlighted, as an elevated expression of APOBEC3s may provide a reason for aberrant cancer-inducing somatic mutations in human papilloma virus (HPV) [75–77] and HBV [78] infections, as well as an extensive range of other tumor types [73, 79, 80], even in the context of inflammation [81].

In fact, A3s strongly inhibit various types of exogenous viruses, including herpesvirus, parvovirus, papillomavirus and hepadnavirus [7, 82–84]. Sheehy et al. (2002) isolated a gene that restricts HIV-1 replication, identified as APOBEC3G [5]. In HIV-1 and other viruses, the virion infectivity factor (Vif) is a potent regulator of virus infection and replication and is consequently essential for pathogenic infections in vivo [85–89]. Vif interacts with A3G, triggers the ubiquitination and degradation of A3G via the proteasomal pathway, by binding A3G and a Cullin5-ElonginBC E3 ubiquitin ligase complex which results in the proteasomal degradation of A3G. Therefore, Vif is required during viral replication to inactivate the host cell antiviral factor A3G [90]. Indeed, the presence of a mutant Vif results in a failure to bind A3G, which in turn results in A3G incorporation into assembling virions with loss of viral infectivity [90].

A3 proteins also inhibit the mobilization of endogenous retroviruses and other retroelements, such as Alu and L1. For instance, Esnault and colleagues (2005) demonstrated that A3G can interfere with the mobilization of murine ERV elements, such as IAP and MusD, by inducing G-to-A hypermutations in the proviral DNA plus strand [91]. In recent years, most A3 family members have been shown to be able to counteract the activity of Alus and L1s in humans and primates, both in the nucleus and in the cytoplasm. For instance, A3G is able to repress Alu retrotransposition without interacting directly with L1 [92, 93], in fact A3G can inhibit L1-dependent retrotransposition by sequestering Alu RNAs in the cytoplasm, therefore being away from the nuclear L1’s enzymatic machinery. Different A3 proteins have diverse cellular localization patterns: A3A/C/H act both in the cytoplasm and in the nucleus; A3B only in the nucleus; A3D/F/G are active in the cytoplasm [94]. Given these critical functions, it is no surprise that the A3 family is being studied in the context of cancer, antiviral and immune-related drug discovery [95–98].

### The evolutionary arms race between APOBEC3 and retrotransposons

The evolutionary arms race [99] is an ongoing struggle between competing sets of co-evolving genes, phenotypic/behavioral traits or species, that develop escalating adaptations and counter-adaptations against each other.

Retrotransposons in humans are counteracted by different mechanisms, for example the Piwi-interacting RNA (piRNA) pathway and the Krüppel-associated box zinc finger (KRAB-ZNF) proteins (reviewed in [33]), which are able to repress TEs mobilization and expression. In a similar way, some components of the APOBEC3 gene cluster are involved in the control of retrotransposons. Indeed, the rapid co-evolution between the A3 locus and different retroviruses, and the positive selection acting on A3 genes are signals of the continuous arms race that characterized A3s, viruses and retroelements [61, 100, 101].

First discovered by Sheehy and colleagues as a defense against HIV-1 virus [5], A3G is able to repress ERVs mobilization in both mouse and human cells, by inducing G-to-A hypermutations in the nascent DNA of ERV elements, such as IAP and MusD in mice and HERV in humans [91]. Therefore, by editing viral genetic material, it provides an ancestral wide cellular defense against endogenous and exogenous invaders.

Other proteins of the A3 family can counteract LTR retrotransposons’ activity: A3A and A3B. A3B acts similarly to A3G, by specifically interacting with the ERV *Gag* protein in co-expressing cells and inducing extensive editing of ERV reverse transcripts [102]. On the contrary, A3A, which can restrict ERVs in human cells by 100-fold (compared to a fourfold inhibition of these elements by A3G), fails to package detectably into ERV virus-like particles and does not edit ERV reverse transcripts [102].

Inhibition of L1 by A3 occurs at the post-transcriptional level by a deamination-dependent or independent mechanism. The most active enzyme (with respect to L1) A3A has deaminase activity and converts C-to-U in the first strand of the L1 cDNA transcript. As a result of such modification, the deamination of transiently exposed DNA leads to the truncation/abortion of retrotransposition [103]. A different mechanism has been identified for A3C and A3D: acting by a deamination-independent mechanism, the enzyme blocks the L1 reverse transcription reaction by interacting with the L1 complex of ribonucleoprotein (RNP) and ORF1 in the cell cytoplasm [104, 105].

Recently, Uriu and colleagues (2021) investigated the evolutionary forces that drove the generation of the youngest A3 members, i.e. A3B and A3D/F. Notably, the invasion of LINE-1 and Alu peaked around the age of the common ancestor of Catarrhini (29 to 43 Mya), concurrently with the generation of A3B and the duplication of A3D/F, suggesting that the origin of these A3 genes in the common ancestor of Catarrhini could be driven by the invasion of LINE-1 and Alu [64]. The same Authors suggest that the origin of A3G dates back to the age of the common ancestor of Simiiformes (67–43 Mya), when there was an invasion of ERV elements. Indeed, A3B potently suppresses the growth of LINE-1 [106–108], whereas A3F inhibits the replication of *vif*-deleted HIV-1 [109], HERV-K [110] and LINE-1 [106]. Altogether, these findings suggest that retrotransposons invasion in the common ancestor of Catarrhini and Simiiformes was a driving force of the powerful co-evolution between TEs and A3 proteins [64].

Interestingly, DNA editing of retrotransposons has been proposed to be a source of genome evolution, in fact DNA editing by APOBEC3 can induce many mutations in a single event. That way, a given element could change to such an extent that its evolutionary trajectory could be altered [66]. With the help of new mutations, retrotransposons’ sequences can vary significantly, and these elements can acquire new and diverse functions in the host genomes. For instance, they can still play a functional role as exapted enhancers or transcriptional start sites [111–114], by inserting Transcription Factor Binding Sites (TFBS) [115, 116] or by acting as novel RNA genes such as long non-coding RNAs (lnc-RNAs) [117]. TEs can also affect translation regulation when transcribed within a mRNA and contribute to protein-coding regions both at the transcript and the protein level, and some TE-encoded proteins have been domesticated and are part of host genes [118]. Moreover, TEs can be involved in the generation of genes and pseudogenes [119–121] and can generate diversity through active transposition in germline cells, which can create novel insertions that are capable of being inherited, thereby generating human-specific polymorphisms. TEs also play key roles in embryogenesis [122–124], speciation [125, 126] and possibly neurogenesis [127–129].

Carmi and colleagues (2011) found many pairs of retrotransposons containing long clusters of G-to-A mutations that cannot be attributed to random mutagenesis and demonstrated that these clusters, which they found across different mammalian genomes and retrotransposon families, are the hallmark of APOBEC3 activity, suggesting a potential mechanism for retrotransposon domestication [130]. Therefore, DNA editing can help to explain how some retrotransposons have acquired such a diverse collection of functions in primate genomes [130].

### Emerging perspectives

Located on human chromosome 22, the APOBEC3 family of deaminase proteins has a wide range of functions, from tumor progression to viruses and retrotransposons restriction.

In this review, we discussed the different mechanisms by which A3 genes inhibit retrotransposons proliferation, by inducing C-to-U or G-to-A hypermutations in the nascent DNA or by interacting with the L1 complex of RNP and ORF1 in the cell cytoplasm.

The origin of the APOBEC3 gene cluster is an extraordinary example of coevolution between a defense mechanism and its counterpart: different A3 genes appeared by duplications, fusions and rearrangements in primates, and such events happened concurrently with the invasion of some retrotransposons, most notably ERV and L1 (Fig. 2). Indeed, a strong evolutionary arms race shaped the evolution of A3 genes and retrotransposons in primates and *Homo*. Diversification and functional differentiation of antiviral genes has led to the establishment of species-specific antiviral defenses, such as that of APOBEC3, which plays a pivotal role in regulating cross-species viral transmission [76]. In summary, the defensive roles of A3 genes are attributable to their rapid and complicated evolution, driven by retrotransposons.

Karagianni and colleagues (2022) have recently suggested that RNA editing is an emerging mechanism in disease development, displaying common and disease-specific patterns, in the context of neuropsychiatric and neurodegenerative disorders [131]. APOBEC3-driven RNA editing is responsible for alternative splicing, regulation, degradation, and secondary structure changes that directly affect nucleic acid functions in the brain [131]. As highlighted previously, A3s are involved in retrotransposons inhibition and, although the mechanistic details of the functional and evolutionary impact of retrotransposons in the brain and nervous system are still unknown, an increasing bulk of data suggests that TEs play a role in the development of the CNS (reviewed in [132–134]) and contribute to neurological disorders (as recently reviewed in [135–137]). Commonly edited RNAs represent potential disease-associated targets for therapeutic and diagnostic values [131]: indeed, a recent work by Macciardi and colleagues (2022) showed that a strong dysregulation in TEs expression is associated with different stages of Alzheimer’s disease development, providing clues on the use of expression profiles as potential predictors of the disease [138]. These findings have major implications for understanding the neuroplasticity of the brain, which probably had a remarkable impact on brain evolution in mammals, especially in Hominids, and could contribute to vulnerability to neurological disorders.

During mammalian embryonic development, retrotransposons are expressed at different levels and play essential roles in embryonic stem cells (ESC) differentiation and pre-implantation embryos, as suggested by several recent publications [118, 123, 124, 139]. Moreover, it is proposed that mutator proteins such as the APOBEC superfamily may interfere with retrotransposon expression patterns to determine different levels of TEs activity in different cell types [107, 108, 118]. Indeed, it is suggested that A3B is highly expressed in human pluripotent stem cells, making LINE-1 silencing more efficient in the early stages of cell differentiation [108]. This is in line with experimental findings that retrotransposons (both LTR and non-LTR) are predominantly active in human embryos at the 8-cell stage and are down-regulated following whole-genome activation [140, 141]. Furthermore, it is reported that all APOBEC3 proteins seem to be able to act as inhibitors of LINE-1 retrotransposons [142–145], while Alu elements are particularly restricted by A3F and A3G, sometimes in macromolecular complexes [92, 93, 146]. These observations point towards an essential contribution of APOBECs as modulators of TEs expression across embryonic developmental trajectories, although further studies are needed to elucidate the link between A3 proteins, retrotransposons, and developmental processes.

## Conclusions

Retrotransposons are endogenous genetic elements with the ability to move around in the genome, and because of their high mutagenic potential the majority of TEs have been faced with negative selection and are counteracted by numerous mechanisms. In primates and humans, A3 genes probably arose in the context of a strong evolutionary arms race between retrotransposons and their hosts, leading to the expansion of this family of mutator proteins, which eventually became one of the strongest host defense mechanisms. The functional relationships between exogenous viral elements and the A3 family already suggested a similar association; however, several recent studies have pinpointed the positive impact of the non-coding genome on human and primate evolution through the regulation of gene expression (for example, during embryonic development). This, in turn, is paving the way for new discoveries around the evolutionary association between retrotransposons and A3 proteins, especially in the context of primate speciation. Interestingly, one of the peculiarities of primates is related to brain development, especially in the Hominoidea lineage. Indeed, retrotransposons contributed to the evolution of the CNS throughout primate phylogeny, exerting a remarkable influence on the tradeoff between brain physiology and pathological conditions in humans. In conclusion, the competition between retrotransposons and APOBEC3 genes has not only led to the development of a diversified immune defense mechanism but has also contributed to the evolutionary relationships among the primate species that are currently known.

## Data Availability

Data sharing not applicable to this article as no datasets were generated or analysed during the current study.

## Abbreviations

A3: APOBEC3 genes
cDNA: Circular DNA
CD: Cytosine deaminase
CNS: Central nervous system
ERV: Endogenous retrovirus
ESC: Embryonic stem cells
FVA: FRAM (Free Right Alu Monomer)-VNTR-Alu
HBV: Hepatitis B virus
HERV: Human endogenous retrovirus
HIV: Human immunodeficiency virus
HPV: Human papillomavirus
KRAB-ZNF: Krüppel-associated box zinc finger
LAVA: L1-Alu-VNTR-Alu
LINE: Long interspersed nuclear element
L1: LINE-1 retrotransposon
NWM: New world monkeys
ORF: Open reading frame
OWM: Old world monkeys
piRNA: Piwi-interacting RNA
PVA: PTGR2-VNTR-Alu
RNP: Ribonucleoprotein
SINE: Short interspersed nuclear element
SIV: Simian immunodeficiency virus
SVA: SINE-VNTR-Alu
TEs: Transposable elements
UTR: Untranslated region
Vif: Virion infectivity factor
VNTR: Variable number tandem repeats
